# Impact of magnesium:calcium ratio on calcification of the aortic wall

**DOI:** 10.1371/journal.pone.0178872

**Published:** 2017-06-01

**Authors:** Ricardo Villa-Bellosta

**Affiliations:** 1 Fundación Instituto de Investigación Sanitaria, Fundación Jiménez Díaz (FIIS-FJD), Avenida Reyes Católicos 2, Madrid, Madrid, Spain; 2 Spanish Biomedical Research Network in Diabetes and Associated Metabolic Disorders (CIBERDEM), Madrid, Spain; Universita degli Studi di Bari Aldo Moro, ITALY

## Abstract

**Objective:**

An inverse relationship between serum magnesium concentration and vascular calcification has been reported following observational clinical studies. Moreover, several studies have been suggesting a protective effect of magnesium on the vascular calcification. However, the exact mechanism remains elusive, and investigators have speculated among a myriad of potential actions. The effect of magnesium on calcification of the aortic wall is yet to be investigated. In the present study, the effects of magnesium and calcium on the metabolism of extracellular PPi, the main endogenous inhibitor of vascular calcification, were investigated in the rat aorta.

**Approach and results:**

Calcium and magnesium have antagonist effects on PPi hydrolysis in the aortic wall. *K*_*m*_ and *K*_*i*_ values for PPi hydrolysis in rat aortic rings were 1.1 mmol/L magnesium and 32 μmol/L calcium, respectively, but ATP hydrolysis was not affected with calcium. Calcium deposition in the rat aortic wall dramatically increased when the magnesium concentration was increased (ratio of Mg:Ca = 1:1; 1.5 mmol/L calcium and 1.5 mmol/L magnesium) respect to low magnesium concentration (ratio Mg:Ca = 1:3, 1.5 mmol/L calcium and 0.75 mmol/L magnesium).

**Conclusion:**

Data from observational clinical studies showing that the serum magnesium concentration is inversely correlated with vascular calcification could be reinterpreted as a compensatory regulatory mechanism that reduces both PPi hydrolysis and vascular calcification. The impact of magnesium in vascular calcification in humans could be studied in association with calcium levels, for example, as the magnesium:calcium ratio.

## Introduction

Calcium-phosphate deposition in the vessel system (vascular calcification) is observed during aging, diabetes and chronic kidney disease. Extracellular fluids are supersaturated with phosphate and calcium ions, which induces the spontaneous formation of calcium-phosphate crystals (CPCs), along with their deposition in the aortic wall and other soft tissues[1,2]. Inhibitors are essential for treating and/or preventing these disease symptoms. Current inhibitors include large proteins such as matrix gla protein, osteopontin and fetuin A, and low molecular weight molecules such as pyrophosphate[3]. Extracellular pyrophosphate (PPi) is a potent inhibitor of vascular calcification, which directly inhibits CPC formation *in vitro* and *in vivo*[4–8]. Recent studies showed that endogenous production of PPi and daily injections of exogenous PPi prevent medial vascular calcification in several murine models[4,8–10].

Extracellular PPi is produced by hydrolysis of ATP via ectonucleotide pyrophosphatase/phosphodiesterase 1(eNPP1)[11]. Mutation of eNPP1 results in generalised arterial calcification of infancy (GACI), which is characterised by calcification of arteries[12], and eNPP1-null mice also develop ectopic artery calcification[13,14]. Moreover, PPi is degraded to Pi enzymatically by tissue non-specific alkaline phosphatase (TNAP). Over-expression of TNAP in cells is sufficient to cause medial vascular calcification in aortic rings *ex vivo*[11], and addition of alkaline phosphatase to the culture medium causes matrix calcification. Moreover, TNAP expression is increased in medial vascular calcification models such as uremic rats, or in a mouse model of Hutchinson-Gilford Progeria Syndrome[4,15]. In addition, alkaline phosphatase activity is increased during dialysis therapy[16].

According to several studies, calcium is more of a determinant for CPC formation than is phosphate[1,17]. For example, CPCs are not formed when the concentration of phosphate is high but the calcium concentration is low. By contrast, CPCs are readily formed when the calcium concentration is high, even when the phosphate concentration is low[1]. Hydroxyapatite, for which the molecular formula is (Ca_10_(PO_4_)_6_(OH)), is the main type of CPC found in vascular calcification and bone, whereas octocalcium phosphate (Ca_8_(PO_4_)_6_.5H_2_O) and amorphous calcium phosphates (Ca_9_(PO_4_)_6_.nH_2_O) are present in calcified tissues. Moreover, a magnesium-substituted form of CPCs (Whitlockite; Ca_9_Mg(PO_4_)_6_(OH)) found in calcified tissues has been implicated in several diseases. The deposition of these CPCs, both *in vitro* and *in vivo*, takes place on high glycine content extracellular matrix proteins such as elastin and collagen[1]. According to the charge neutralisation theory of calcification[18], the high glycine content of these matrix proteins favours the formation of β-turns that are known to interact with calcium ions and influence the growth of CPCs.

Several studies indicate the protective effects of magnesium on vascular calcification, due to the antagonistic effects of magnesium on calcium functions including hydroxyapatite formation and calcium transport into cells[19,20]. Since magnesium and calcium are macroelements that are present in extracellular fluids in relatively high concentrations (mmol/L range), the impact of magnesium and calcium ions on extracellular PPi metabolism and its implication on calcification of the aortic wall were investigated for the first time in the present study.

## Materials and methods

### Statistical analyses

**Fig 1:**
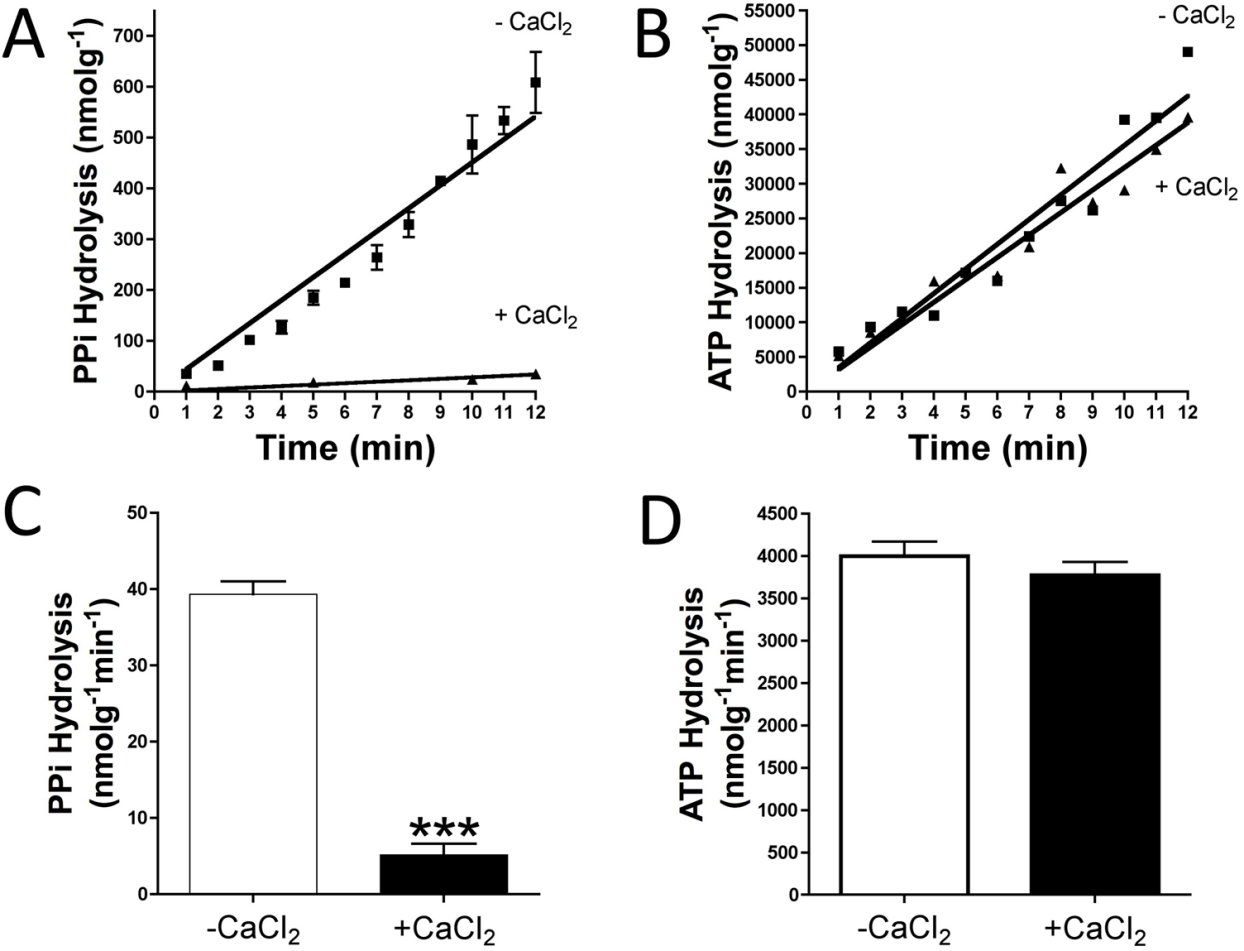
Impact of calcium on the hydrolysis of pyrophosphate and ATP. Rat aortic ring tissue samples were incubated *ex vivo* in sHBSS (supplemented with 0.9 mmol/L magnesium) with 1 mmol/L calcium (+CaCl_2_) or without calcium (-CaCl_2_). (**A**) Representative time course for the hydrolysis of 5 μmol/L PPi (and 10 μCi/mL ^32^PPi as radiotracer) measured by Pi release (^32^Pi). (**B**) Representative time course of the hydrolysis of 1 μmol/L ATP (and 10 μCi/mL [γ^32^P]ATP as radiotracer) measured by Pi release (γ^32^Pi). Results in (**C**) and (**D**) are means ± SEM, after 10 min of incuation, of three independent experiments, with a total of 20 rings per condition (10 different rats). Student’s t-test was used for statistical analysis. ***p <0.001.

**Fig 2:**
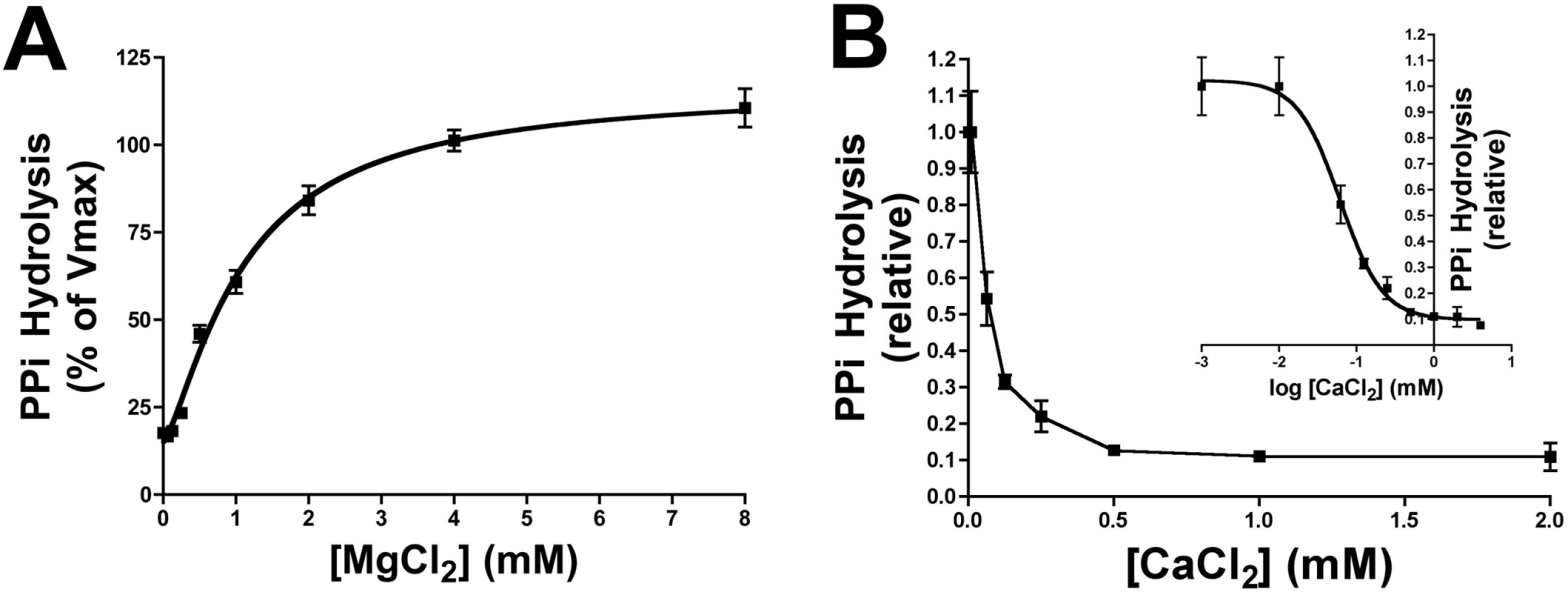
Kinetic characterisation of calcium and magnesium on pyrophosphate hydrolysis. Rat aortic rings were incubated *ex vivo* in HBSS containing the indicated amount of magnesium and calcium. (**A**) Michaelian saturation curves were plotted to determinate the *K*_*m*_ value of magnesium for PPi hydrolysis. HBSS contained 5 μmol/L PPi and 10 μCi/mL ^32^PPi as radiotracer. (**B**) Competitive inhibition of PPi hydrolysis by calcium; inset, logarithmic transformation of calcium concentration. HBSS contained 1.1 mmol/L magnesium, 5 μmol/L PPi and 10 μCi/mL ^32^PPi as radiotracer. PPi hydrolysis was measured by Pi release. Pi was separated from PPi using the molybdate method as explained in the methods section. Results are represented as means ± SEM of three independent experiments with a total of 6, 7 or 13 rings per point (see S2 Table) and 26 rats in total (A) and two independent experiments with a total of 6 rings per point (see S3 Table) and 12 rats in total (B).

**Fig 3:**
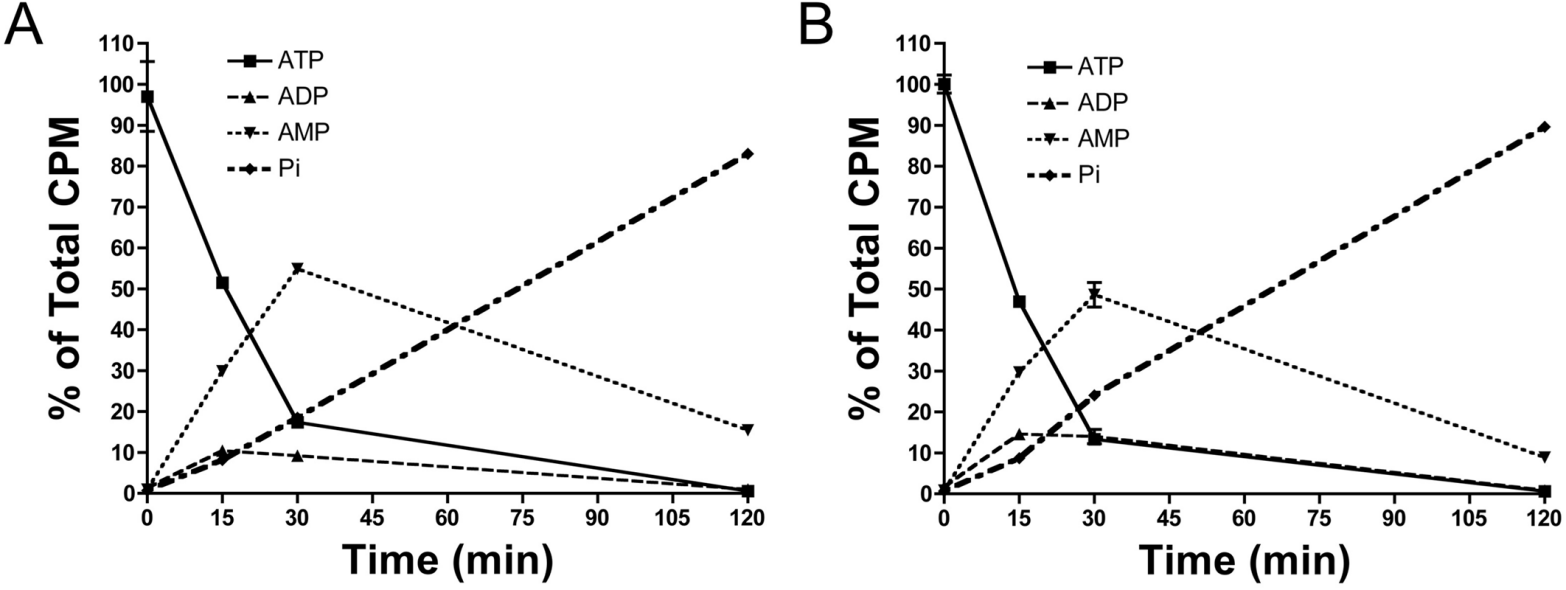
Impact of calcium on ATP hydrolysis. Rat aortic rings were incubated *ex vivo* in sHBSS (supplemented with 1 mmol/L magnesium) and with 1 mmol/L calcium (**A**) or without calcium (**B**). Hydrolysis of 1 μmol/L ATP (and 10 μCi/mL [α^32^P]ATP as radiotracer) showed ADP, AMP and α-Pi production in the indicated times, following separation by thin layer chromatography as described in the methods section. Results are means ± SEM of two independent experiments, with a total of 6 rings per condition (6 different rats). The same ring was used to analyze the hydrolysis of ATP in the absence or presence of calcium.

**Fig 4:**
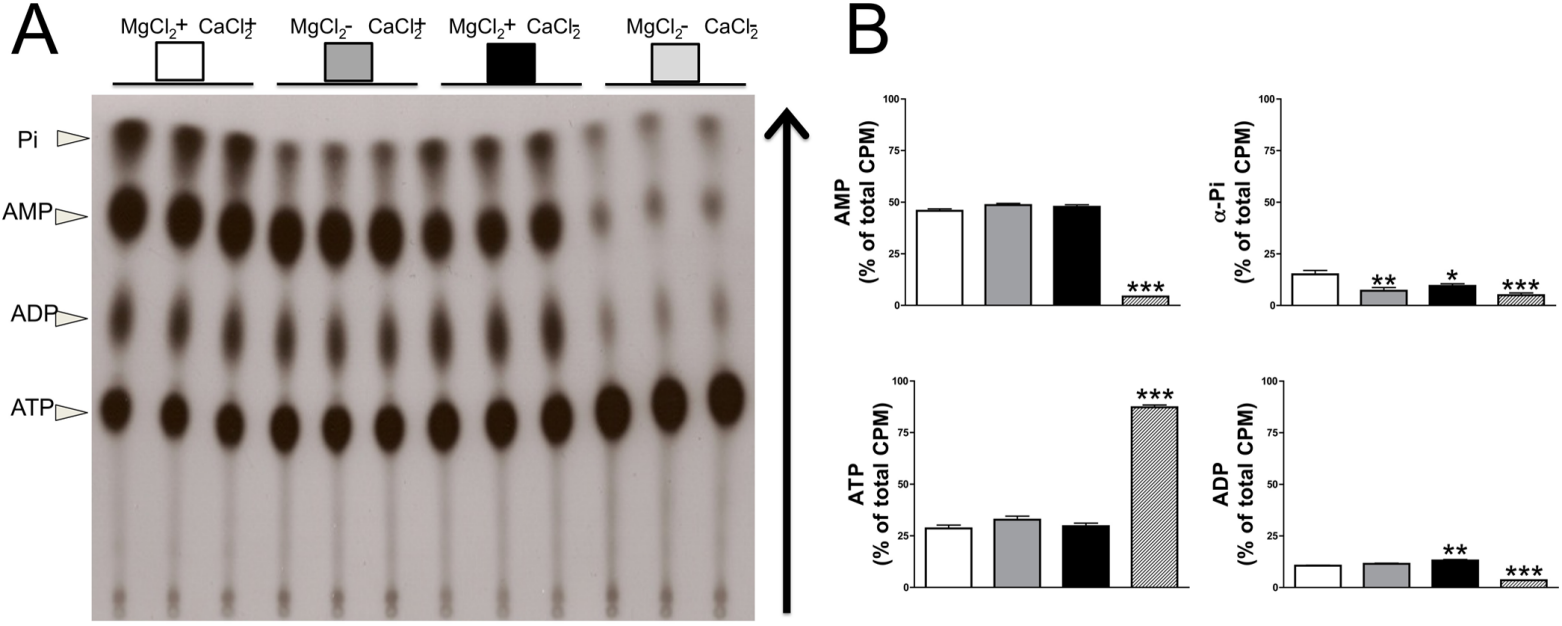
Impact of calcium and magnesium on ATP hydrolysis products. Rat aortic rings were incubated *ex vivo* in HBSS supplemented with 1 mmol/L calcium and 1 mmol/L magnesium as indicated. After 30 min of incubation with 1 μmol/L ATP (and 10 μCi/mL [α^32^P]ATP as radiotracer), ADP, AMP and Pi products were separated as described in the methods section. (**A**) Representative thin layer chromatography showing ADP, AMP and Pi products. (**B**) Quantification of ATP ([α^32^P]ATP), ADP ([α^32^P]ADP), AMP ([α^32^P]AMP) and Pi (α^32^Pi) products obtained from ATP hydrolysis. Results are represented as means ± SEM of three independent experiments with a total of 9 rings from 9 different rats (see S4 Table). The same ring was used to analyze the hydrolysis of ATP in the four conditions. Repeated measured ANOVA and Tukey’s multiple comparison tests were used for statistical analysis. Presence of magnesium and calcium was used as reference. **p <0.01; ***p <0.001.

**Fig 5:**
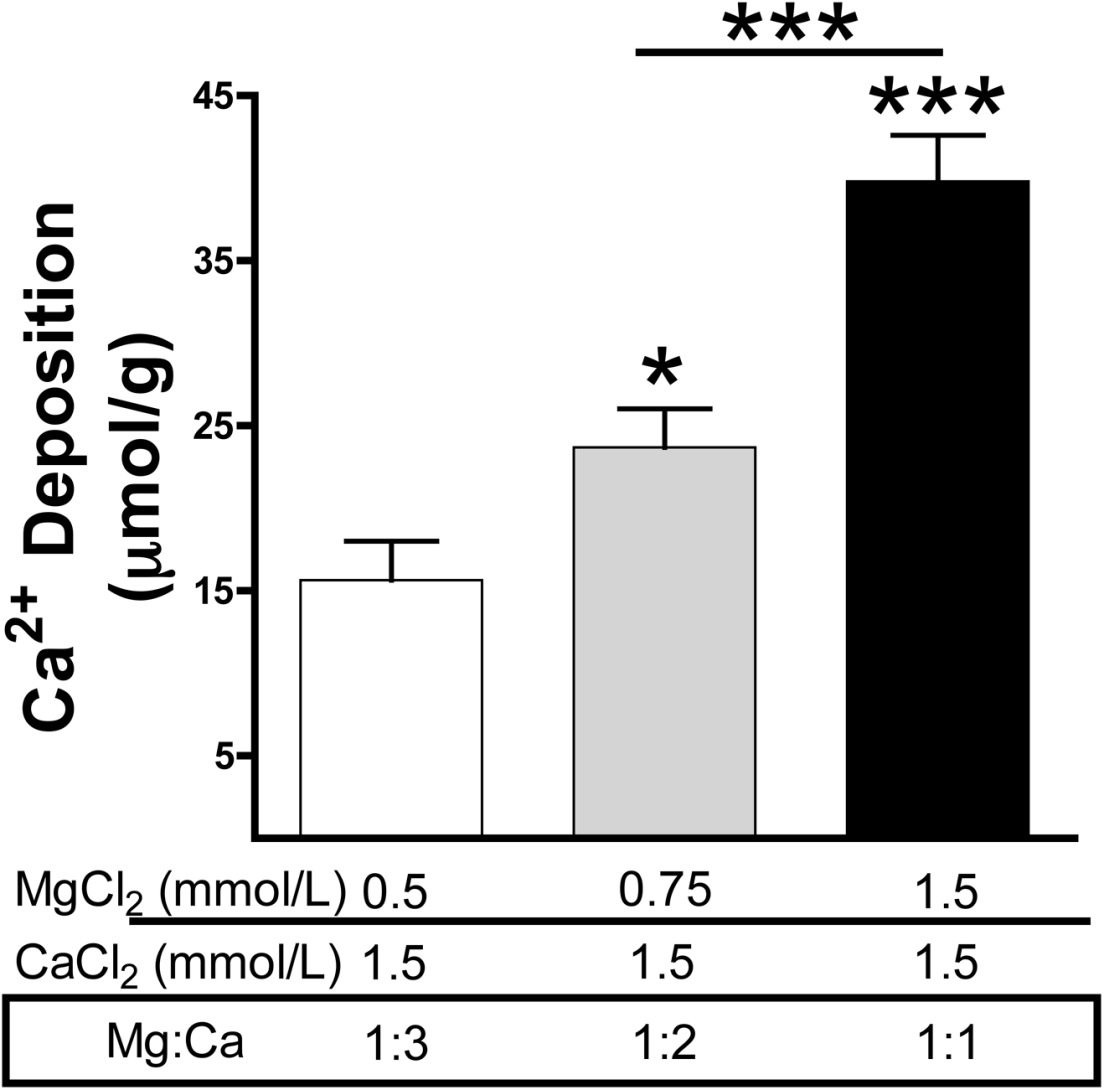
Impact of calcium and magnesium on aortic wall calcification. Rat aortic rings were incubated *ex vivo* in DMEM supplemented with the indicated concentration of magnesium and calcium. The medium was replaced every day and contained 45-calcium as a radiotracer. After 7 days of incubation, aortic rings were dried and radioactivity was measured by liquid scintillation counting. Results are represented as means ± SEM of two independent experiments, with a total of 14 rings per condition and 11 rats in total (see S5 Table). One-way ANOVA and Tukey’s multiple comparison test were used for statistical analysis. *p <0.05; ***p <0.001.

Results in Figs 1, 2, 3, 4 and 5 are presented as means and standard errors of the mean (SEM). In Fig 1C and 1D, the Student’s t-test was used for statistical analysis. In Fig 4B Repeated Measures ANOVA and Tukey’s multiple comparison test were used. In Fig 5, one-way ANOVA and Tukey’s multiple comparison test were used. Statistical significance was determined with GraphPad Prism 5 and assigned at *p*<0.05.

### Animals

Male Sprague-Dawley rats (8–12 week old) were obtained from Charles River Laboratories (France). The protocol was approved by the FIIS-FJD (Fundación Instituto de Investigación Sanitaria, Fundación Jiménez Díaz) ethics committee and conformed to directive 2010/63EU and recommendation 2007/526/EC regarding the protection of animals used for experimental and other scientific purposes, enforced in Spanish law under RD1201/2005.

### Aorta isolation and calcification assay

Rats were euthanised by carbon dioxide inhalation and aorta tissue was perfused with saline and removed according to previously published protocols[21]. For calcification assays, aortic rings were cultured *ex vivo* (37°C, 5%CO_2_) in Minimum Essential Medium Eagle (MEM Media, Gibco, Paisley, United Kingdom) containing the indicated calcium and magnesium concentration, as previously described[11]. The medium was replaced every day and contained 45-calcium (Perkin Elmer, Boston, EE.UU.) as a radiotracer. After 7 days of incubation, aortic rings were dried and radioactivity was measured by liquid scintillation counting (Perkin Elmer Tri-Carb 2810TR).

Previous studies have demonstrated by specific stainings (alizarin red and von Kossa) that aortas from young rats are not calcified. Furthermore, the experiment with 45-calcium detects calcium incorporated from the beginning of the experiment so it does not account for the possible calcium present previously. However, *in vivo* imaging techniques (such as microCT) could be also useful to confirm that calcifications do not exist before selecting rats for the experiment.

### PPi metabolism

For PPi/ATP hydrolysis experiments, aortic rings were cultured *ex vivo*[11,22] in Hanks’ Balanced Salt Solution (HBSS, no calcium and no magnesium, 14185–052, Gibco) supplemented with the indicated amount of calcium chloride (499609, Sigma-Aldrich, St. Louis, MO) or magnesium chloride (M8266, Sigma-Aldrich) to generate supplemented HBSS (sHBSS). To analyse the products released during ATP hydrolysis, aortic rings were incubated in sHBSS containing ATP and [α^32^P]ATP (Perkin Elmer) at a final concentration of 1 μmol/L and 10 μCi/mL, respectively. After the indicated time (as shown in the figures), production of ADP, AMP, Pi and ATP was determined by chromatography on PEI-cellulose plates (50488-25EA-F; Sigma-Aldrich) developed with 650 mmol/L K_2_HPO_4_ (P5655, Sigma-Aldrich) pH3, as previously described[4,11,16,22]. After radiography, spots were excised and counted by liquid scintillation (Ultima Gold^TM^, 6013329; Perkin Elmer). To analyse kinetic parameters for PPi/ATP hydrolysis, aortic ring tissue samples were incubated with 5 μmol/L PPi or 1 μmol/L ATP in sHBSS containing ^32^PPi or [γ^32^P]ATP (Perkin Elmer) as radiotracer (10 μCi/mL), respectively. Orthophosphate was separated from ATP and PPi as previously described. Briefly^11^, 20 μL of sample was mixed with 400 μL of ammonium molybdate (to bind the orthophosphate; 09913, Sigma-Aldrich) and 0.75 mol/L sulphuric acid (258105, Sigma-Aldrich). Samples were then extracted with 800μL of isobutanol/petroleum ether (4:1) to separate the phosphomolybdate from PPi and ATP (refs. 77379 and 360465 for petroleum ether and isobutanol, respectively; Sigma-Aldrich). Next, 400 μL of the organic phase containing phosphomolybdate was removed and subjected to radioactivity counting. In the experiments shown in Fig 3, the same ring was used to analyze the hydrolysis (^32^PPi or α^32^P]ATP) in the absence or presence of calcium. In the experiments shown in Fig 4, the same aortic rings were used for the four conditions. Rings were washed five times in HBSS without calcium and without magnesium before performing the experiment with each condition. In all experiments, aortic rings were also normalized by dry weight (g) aorta.

### Kinetic analyses

Magnesium saturation kinetics for PPi hydrolysis were fitted to the Hill equation (equation 1; V = [V_max_ S^n^ / (K_m_^n^ + S^n^)] + V_0_), were **V** is the velocity of PPi hydrolysis, **V**_**max**_ is the maximal velocity or capacity of PPi hydrolysis, **S** is the concentration of magnesium ([Mg]), **n** is the Hill coefficient; ***K***_***m***_is the affinity constant and V_0_the velocity when [Mg] equals zero.

The mean inhibitory concentration (**IC**_**50**_) was calculated by nonlinear regression using the one-site competition equation (1), V = Bottom+(Top-Bottom)/[1+10^(S-logIC50)^], where Top refers to the velocity of Pi released in the absence of inhibitor (calcium), and Bottom refers to the maximal inhibition. The inhibition constant, ***K***_***i***_, was then calculated indirectly using equation 3, *K*_*i*_ = IC_50_/[1+(S/*K*_*m*_)], where **S** is the constant concentration of magnesium, and ***K***_***m***_ is the affinity constant of magnesium (according to equation 1).

## Results

### Effects of calcium and magnesium on PPi degradation

To analyse the role of calcium in extracellular PPi metabolism, we first investigated the impact of calcium on PPi degradation. Fig 1A and 1B shows a representative time course of the hydrolysis of 5 μmol/L PPi or 1 μmol/L ATP, respectively. Moreover, Fig 1C shows that PPi hydrolysis in rat aortic rings was 7,6-fold less in the presence of calcium (5.11±0.58 nmol*g^-1^min^-1^) than in the absence of calcium (38.75±1.56 nmol*g^-1^min^-1^). By contrast, ATP hydrolysis (Fig 1D and S1 Table) was unaltered in the absence or presence of calcium (4001±169 nmol*g^-1^min^-1^ vs. 3775±154.7 nmol*g^-1^min^-1^).

PPi hydrolysis in the aorta wall in the absence of calcium and in the presence of different concentrations of magnesium was then tested. As shown in Fig 2A, hydrolysis of PPi increased with increasing magnesium concentration in a dose-dependent manner, with a Michaelis-Menten constant (*K*_*m*_) of 1.1 mmol/L magnesium, a velocity in the absence of magnesium (*V*_*o*_) of 15.20% and a Hill coefficient (*n*) of 1.36. Moreover, PPi hydrolysis with 1 mmol/L magnesium was inhibited by calcium in a dose-dependent manner. As shown in Fig 2B, the IC_50_ value was 61 μmol/L calcium, which corresponds to an inhibitory constant (*K*_*i*_) of 32 μmol/L.

### Effects of calcium and magnesium on PPi synthesis

Fig 3 shows the product of [α^32^P]ATP degradation in the aortic wall in the presence (Fig 3A) or absence (Fig 3B) of calcium. In both cases, 1 μmol/L ATP was completely degraded after 1 h, and no differences were observed in the production of AMP over time. Moreover, the absence of both magnesium and calcium completely impaired ATP hydrolysis in the aortic wall (Fig 4A), as can be seen in Fig 4B with the presence of most of ATP and the absence of its degradation products (ADP, AMP and α-Pi). The absence of magnesium (in presence of calcium) impaired the hydrolysis of AMP and thus the formation of Ado and Pi (Fig 4A and 4B; α-Pi), the main reaction of 5`-ectonucleotidase (5NT, also known as CD73). In addition, in the presence of magnesium, the Pi released by [α^32^P]ATP hydrolysis significantly decreased in the absence of calcium compared to its presence (Fig 4B; α-Pi). Finally, in the presence of both calcium and magnesium, all product of [α^32^P]ATP hydrolysis (ADP, AMP and α-Pi) were produced (see S4 Table).

### Effect of the magnesium:calcium ratio on aortic calcification

Since both calcium and magnesium are present in the mmol/L range, and since they have opposite effects on PPi hydrolysis (the main inhibitor of CPC formation), was evaluated the role of the magnesium:calcium ratio on vascular calcification. As shown in Fig 5, calcium was deposited on rat aortic rings cultured *ex vivo*, and deposition was greater (4-fold; *p*<0.001) with 1.5 mmol/L Ca^2+^ and 1.5 mmol/L Mg^2+^ (Mg:Ca ratio = 1:1) than with1.5 mmol/L Ca^2+^and 0.5 mmol/L Mg^2+^ (Mg:Ca ratio = 1:3).This finding further indicates that increasing the magnesium concentration increases PPi hydrolysis, and therefore stimulates vascular calcification.

## Discussion

An inverse relationship between serum magnesium concentration and vascular calcification has been reported in observational clinical studies[23,24]. For example, Meema et al.[23] reported a magnesium concentration of 2.69 mg/dL vs. 3.02 mg/dL (*p*<0.001) when vascular calcification was and was not observed, respectively. Moreover, Ishimura et al.[24] showed that serum magnesium was significantly lower in patients with vascular calcification than in those without (2.69 mg/dL vs. 2.78 mg/dL, *p*<0.05).

Several studies reported protective effects of magnesium on vascular calcification[19,20]. However, the picture remains complicated because magnesium homeostasis could potentially interact with calcium and phosphate homeostasis. For example, an inverse relationship between magnesium and PTH has been described[25,26]. Turgut et al.[19] showed that magnesium supplementation in patients receiving haemodialysis displayed increased plasmatic magnesium after 2 months (2.5 vs. 2.69 mg/dL, *p* = 0,001), with an associated reduction in PTH level (392.7 vs. 214.8 pg/mL, *p* = 0.003), and a mild reduction in calcium level (9.4 vs. 9.2 mg/dL). Moreover, Gorgels et al.[20] showed that dietary magnesium reduces the extent of calcification in the heart (determined by μCT) in comparison with calcium supplementation in a mouse model of pseudoxanthoma elasticum. Interestingly, in this study[20] supplementation of magnesium was administered along with calcium, and serum calcium levels were significantly reduced when both calcium and magnesium were supplemented, compared with calcium supplementation alone (2.85 vs. 2.96 mg/dL, *p* <0.05).

Magnesium is considered a natural antagonist of calcium, the main inducer of vascular calcification. The results of the present study demonstrate, for first time, that calcium and magnesium have an antagonistic effect on hydrolysis of PPi in the aortic wall. Magnesium and calcium promote and inhibit PPi hydrolysis, respectively (Fig 2). By contrast, the presence of magnesium or calcium had no effect on PPi synthesis from ATP hydrolysis. Interestingly, *K*_*m*_ and *K*_*i*_ values for PPi hydrolysis in rat aortic rings were 1.1 mmol/L magnesium and 32 μmol/L calcium. Since the normal range of magnesium is 0.7–1.1 mmol/L (1.7–2.6 mg/dL), we can hypothesise that variation in the magnesium concentration may affect PPi hydrolysis and aortic wall calcification. Since calcium is normally in the mmol/L range (60-fold higher than the *K*_*i*_ value), the effect of calcium on the inhibition of PPi hydrolysis should be explored in association with the magnesium concentration. Indeed, to understand the impact of calcium and magnesium on vascular calcification, both should be considered together rather than separately. The magnesium:calcium ratio may therefore prove to be a useful parameter during clinical and experimental studies. For example, this study revealed greater calcium deposition on aortic rings when the Mg:Ca ratio was high, under conditions when the magnesium concentration increases and the calcium concentration remains constant (Fig 5).

Finally, the calcium concentration plays an important role in CPC formation and deposition. However, a recent study excluded a physicochemical role for magnesium in altering CPC growth, composition or structure[27]. An increase in calcium stimulates CPC deposition[1] and, moreover, inhibits PPi hydrolysis (Figs 1 and 2). This may reflect compensatory regulation that increases the availability of PPi, a potent endogenous inhibitor of CPC. The findings of the present study expand our knowledge of PPi biology and the effects of calcium and magnesium on aortic wall calcification.

In conclusion, data from observational clinical studies showing that the serum magnesium concentration is inversely correlated with vascular calcification[23,24] could be reinterpreted as a compensatory regulatory mechanism that reduces both PPi hydrolysis (Fig 2) and vascular calcification (Fig 5). Moreover, data from experimental studies shown a protective effect of magnesium on the vascular calcification by alteration in calcium homeostasis [19, 20, 25, 26] but not be alteration in growth, composition or structure[27]. However, magnesium supplementation could have additional unexplored effect that could compensate the stimulation of PPi degradation. The results suggest that the impact of magnesium in vascular calcification in clinical or experimental studies should be analysed in association with the calcium level, and the magnesium:calcium ratio might serve as a useful parameter in this regard.

## Supporting information

S1 Table Experimental results Fig 1. (PDF)

S2 Table Experimental results Fig 2A. (PDF)

S3 Table Experimental results Fig 2B. (PDF)

S4 Table Experimental results Fig 4B. (PDF)

S5 Table Experimental results Fig 5. (PDF)
