# Supplementary material for: Impact of magnesium:calcium ratio on calcification of the aortic wall
Source: PLoS One. 2017 Jun 1;12(6):e0178872. doi: 10.1371/journal.pone.0178872 (PMC5453594; doi:10.1371/journal.pone.0178872)
Supplement: S1 Table — (PDF) [file pone.0178872.s001.pdf]

S1 Table

PLOS ONE
Impact of magnesium:calcium ratio on calcification of the aortic wall
Ricardo Villa-Bellosta

Fig 1C

PPi Hydrolysis (nmol*g-1*min-1)		
CaCl2 (-)	CaCl2 (+)	
32,01	4,59	Experiment 1
28,62	3,21	
34,16	4,34	
37,03	5,83	
41,18	2,00	
44,14	2,97	
46,99	10,29	Experiment 2
42,92	3,66	
46,07	2,83	
45,71	2,89	
32,68	4,93	
26,89	3,58	
35,89	5,90	Experiment 3
34,70	4,22	
39,75	4,11	
34,40	12,92	
34,33	5,07	
38,07	4,88	
45,14	6,62	
54,36	7,25	

Fig 1D

ATP Hydrolysis (nmol*g-1*min-1)		
CaCl2 (-)	CaCl2 (+)	
6319,41	5648,50	Experiment 1
5094,59	4676,20	
4198,71	3895,82	
2999,28	4353,04	
3745,49	4795,16	
2908,54	3044,45	
3494,82	3261,11	Experiment 2
3757,10	3032,69	
3173,97	3309,29	
4282,95	4170,05	
3918,84	3464,39	
4457,35	3329,37	
3413,78	3186,35	Experiment 3
3692,22	3522,76	
3910,97	4068,00	
4176,57	3230,96	
4394,20	3679,71	
4193,37	3114,39	
4005,56	4092,10	
3878,21	3626,59	
