# Supplementary material for: Impact of magnesium:calcium ratio on calcification of the aortic wall
Source: PLoS One. 2017 Jun 1;12(6):e0178872. doi: 10.1371/journal.pone.0178872 (PMC5453594; doi:10.1371/journal.pone.0178872)
Supplement: S2 Table — (PDF) [file pone.0178872.s002.pdf]

S2 Table

PLOS ONE
Impact of magnesium:calcium ratio on calcification of the aortic wall
Ricardo Villa-Bellosta

Fig 2A

PPi hydrolysis % of Vmax

Experiment 1

[MgCl2] (mmol/L)						
**0**	12,78	17,07	15,86	21,03	15,16	17,79
**0,0625**	19,26	13,47	17,78	17,04	14,00	18,58
**0,125**	15,46	10,86	23,35	10,82	20,93	14,15
**0,25**	19,28	17,03	23,08	19,25	25,29	26,09
**0,5**	27,62	47,40	37,22	58,00	42,81	75,96
**1**	17,10	60,19	32,99	80,59	36,66	82,70
**2**	62,34	83,80	63,75	79,37	67,13	81,12
**4**	102,32	68,55	119,72	90,62	95,53	77,27

Experiment 2

[MgCl2] (mmol/L)				
**0**	11,56	14,13	13,94	16,54
**0,125**	15,06	17,97	17,64	20,53
**0,25**	23,72	16,06	29,09	17,19
**0,5**	23,88	38,76	28,24	43,31
**1**	64,10	55,33	70,07	58,02
**2**	64,08	75,44	71,17	70,03
**4**	99,40	101,25	117,17	90,97
**8**	84,30	126,54	100,37	104,45

Experiment 3

[MgCl2] (mmol/L)			
**0**	26,68	23,14	24,43
**0,125**	18,89	25,11	25,88
**0,25**	19,88	34,83	32,75
**0,5**	50,88	66,86	56,73
**1**	64,00	87,85	81,16
**2**	136,89	142,84	95,99
**4**	138,96	123,39	91,26
**8**	157,40	119,29	81,60
