# Supplementary material for: Impact of magnesium:calcium ratio on calcification of the aortic wall
Source: PLoS One. 2017 Jun 1;12(6):e0178872. doi: 10.1371/journal.pone.0178872 (PMC5453594; doi:10.1371/journal.pone.0178872)
Supplement: S3 Table — (PDF) [file pone.0178872.s003.pdf]

S3 Table

PLOS ONE
Impact of magnesium:calcium ratio on calcification of the aortic wall
Ricardo Villa-Bellosta

Fig 2B

PPi hydrolysis relative

[CaCl2] (mmol/L)			
	Experiment 1		
0,0000	1,19	0,82	0,99
0,0625	0,48	0,58	0,54
0,1250	0,34	0,28	0,31
0,2500	0,16	0,21	0,22
0,5000	0,15	0,11	0,13
1,0000	0,11	0,09	0,11
2,0000	0,08	0,03	0,11
4,0000	0,08	0,06	0,08
	Experiment 2		
0,0000	0,90	1,01	1,07
0,0625	0,73	0,38	0,53
0,1250	0,35	0,28	0,33
0,2500	0,34	0,16	0,22
0,5000	0,11	0,13	0,13
1,0000	0,15	0,09	0,12
2,0000	0,21	0,11	0,13
4,0000	0,06	0,11	0,08
