# Supplementary material for: Impact of magnesium:calcium ratio on calcification of the aortic wall
Source: PLoS One. 2017 Jun 1;12(6):e0178872. doi: 10.1371/journal.pone.0178872 (PMC5453594; doi:10.1371/journal.pone.0178872)
Supplement: S4 Table — (PDF) [file pone.0178872.s004.pdf]

S4 Table

PLOS ONE
Impact of magnesium:calcium ratio on calcification of the aortic wall
Ricardo Villa-Bellosta

Fig 4B

% of Total CPM

Mg(+) Ca(+)	Mg(-) Ca(+)	Mg(+) Ca(-)	Mg(-) Ca(-)	
Experiment 1				
36,32	28,43	28,11	88,41	ATP
25,97	31,83	37,14	91,06	
25,94	38,80	26,22	85,69	
10,29	10,91	14,97	3,72	ADP
11,63	13,45	12,30	3,94	
10,57	12,41	13,00	3,59	
43,64	46,84	45,95	4,05	AMP
44,08	47,11	44,41	4,10	
48,39	47,64	47,19	4,16	
9,75	13,83	10,97	3,82	Pi
18,32	7,62	6,15	0,90	
15,10	1,15	13,59	6,56	
Experiment 2				
24,64	37,96	24,91	81,41	ATP
24,74	30,46	34,32	89,61	
34,51	27,16	25,75	83,98	
10,19	11,19	14,02	3,20	ADP
9,58	10,64	10,24	3,01	
10,80	11,74	13,80	3,39	
44,12	46,66	47,87	4,82	AMP
44,52	47,55	49,97	4,14	
45,96	54,46	48,35	4,50	
21,05	4,19	13,19	10,57	Pi
21,16	11,36	5,47	3,25	
8,73	6,64	12,10	8,12	
Experiment 3				
34,14	29,70	28,46	89,83	ATP
23,50	29,08	34,51	85,13	
27,24	41,64	27,53	89,98	
11,06	10,73	13,69	3,65	ADP
9,51	11,37	11,70	3,23	
11,51	11,08	14,25	3,80	
44,80	49,00	50,36	4,63	AMP
44,52	50,84	43,42	3,83	
52,27	47,16	52,38	4,45	
10,00	10,57	7,48	1,89	Pi
22,47	8,71	10,37	7,81	
8,99	0,11	5,83	1,78	
