# Supplementary material for: Impact of magnesium:calcium ratio on calcification of the aortic wall
Source: PLoS One. 2017 Jun 1;12(6):e0178872. doi: 10.1371/journal.pone.0178872 (PMC5453594; doi:10.1371/journal.pone.0178872)
Supplement: S5 Table — (PDF) [file pone.0178872.s005.pdf]

S5 Table

PLOS ONE
Impact of magnesium:calcium ratio on calcification of the aortic wall
Ricardo Villa-Bellosta

Fig 5

Ca2+ Deposition micromol/g			
7,76	23,23	43,59	Experiment 1
21,28	33,82	40,64	
9,36	16,07	23,70	
13,59	15,71	45,18	
26,10	28,66	45,30	
7,73	23,69	43,39	
21,31	35,02	41,64	
9,43	15,89	23,28	Experiment 2
13,46	17,05	44,94	
26,37	27,66	46,49	
16,93	23,68	42,52	
9,40	26,18	45,72	
15,64	24,76	42,12	
13,52	25,77	39,82	
0,50	0,75	1,50	MgCl2 (mmol/L)
1,50	1,50	1,50	CaCl2 (mmol/L)
